# Needed Improvements in Diabetes Prevention and Management in Brazil

**DOI:** 10.5888/pcd15.180269

**Published:** 2018-12-06

**Authors:** Ana Paula Della Nina de Oliveira, Emanuella Gomes Maia, Flávia Moraes Silva, Ana Paula Bortoletto Martins, Rafael Moreira Claro

**Affiliations:** 1 Masters Program in Nutrition and Health, School of Nursing, Federal University of Minas Gerais, Belo Horizonte (MG), Brazil; 2 Doctoral Program in Nursing, School of Nursing, Federal University of Minas Gerais, Belo Horizonte (MG), Brazil; 3 Nutrition Department, School of Nursing, Federal University of Minas Gerais, Belo Horizonte (MG), Brazil; 4 Center for Epidemiological Research in Nutrition and Health, Faculty of Public Health, University of São Paulo, São Paulo (SP), Brazil

## Abstract

**Introduction:**

Diabetes mellitus is one of the most common chronic diseases worldwide and its prevalence continues to increase. Adherence to good health behaviors provides better control of the disease. This study analyzed trends in the prevalence of diabetes among Brazilian adults between 2006 and 2016 and compared the frequency of key health behaviors between people with and without diabetes.

**Methods:**

We analyzed data from 572,437 Brazilian adults interviewed between 2006 and 2016 by using the Surveillance System of Risk and Protection Factors for Chronic Diseases by Telephone Survey (Vigitel). We used regression models to investigate significant trends in the prevalence of diabetes and differences in adherence to health behaviors between people with and without diabetes.

**Results:**

The prevalence of diabetes increased significantly from 5.5% to 8.9% (*P* <.001), being higher among women, older adults, and those with less than a high school education. The greatest increase was observed among these groups with higher prevalence. People with diabetes had a lower frequency of risk behaviors and a higher frequency of protective behaviors when compared to people without diabetes. The greatest differences were observed in the consumption of soft drinks and sugar-sweetened beverages (9.5% vs 25.0%) and alcoholic beverages (9.0% vs 17.9%), and the smallest differences were related to regular consumption of fruits and vegetables (40.7% vs 34.0%) and meats with excess fat (24.3% vs 32.2%). People with diabetes reported less leisure-time physical activity (PRa, 0.92 [*P* < .001]) and less transport-related physical activity (PRa, 0.84 [*P* <.001]) than those without diabetes.

**Conclusion:**

Diabetes prevalence increased between 2006 and 2016. People with diabetes had better dietary habits than those without diabetes, but still had risk behaviors such as insufficient physical activity.

## Introduction

Diabetes mellitus (diabetes) is a prevalent chronic disease worldwide. Prevalence (1) of diabetes is increasing because of lifestyle changes in such areas as physical inactivity, inadequate food intake, and obesity (2). The International Diabetes Federation estimates that the total number of people with diabetes will increase worldwide from 425 million in 2017 to 629 million in 2045 (3). The estimate for Brazil ranges from 12.5 million to 21.8 million in 2045, ranking the country in fifth place among countries with the highest prevalence of diabetes (3). In this context, the public health system of Brazil has national actions focused on diabetes diagnosis and management (4), but, in 2017, almost half (46.0%) of the adult population with diabetes still had not received a medical diagnosis (3).

Beyond obesity, behavioral factors such as inadequate food intake, sedentary lifestyle, and excessive alcohol consumption (≥5 drinks for men and ≥4 for women in a day) are related to higher diabetes risk (3,5). In this sense, recent evidence indicates an increase in obesity prevalence in Brazil (in both sexes, at all ages, geographic regions, and income levels) (6) and in the consumption of ultraprocessed foods (such as soft drinks, cookies, crackers, and chips) from 18.7% in 1987 to 29.6% in 2009 (7). Almost half of adults (46.0% in 2013) were insufficiently active (8) and 13.7% reported abusive consumption of alcohol (9). Thus, surveillance of these factors in the population is imperative to identify and eliminate risk factors as early as possible in people without diabetes and to minimize them among people with diabetes.

This study analyzed trends in the prevalence of diabetes among Brazilian adults between 2006 and 2016 and compared the frequency of key health behaviors between people with and without diabetes.

## Methods

Data used for this study came from the Surveillance System of Risk and Protection Factors for Chronic Diseases by Telephone Survey (Vigitel) between 2006 and 2016, a total of 572,437 interviews. Vigitel annually interviews a probabilistic sample of the adult population (aged ≥18 years) living in households with at least 1 landline telephone in all 26 Brazilian state capitals and the Federal District (10). The Vigitel sampling process is performed in 2 stages. The first one consists of a sampling of 5,000 landline telephones per city, and the second one is a simple random sample to select 1 adult in each household (10). Each landline telephone selected is called up to 6 times at specific days and hours to verify its eligibility. Nonresidential telephones, out of service telephones, and telephones that are not answered are considered ineligible. Risk and protective factors for noncommunicable diseases can be assessed with a 95% confidence interval (CI) and sample error of 2 percentage points. A maximum of 3 percentage points is expected for estimates by sex (10).

According to the last national Demographic Census in 2010 (11), landline telephones reached 60.8% of households in all 26 Brazilian capitals and the Federal District. Each Vigitel interview is accompanied by a weighting factor aiming to correct the unequal probability of selection of households with more than 1 landline telephone or with more than 1 resident and to equate the distribution of the population interviewed in each city (by sex, age, and schooling) to its entire population (based on intercensal projection for each year) (10).

The data collection team is trained and supervised by technicians from universities and the Ministry of Health. The questions are read by the interviewer from an electronic questionnaire and respondent answers are recorded directly into the database. This process allows identifying the time of the interview, automatically skipping issues that are not applicable because of previous responses, and the continuous feeding into the system database. Further details on the sampling process and data collection employed by Vigitel are available in the annual reports of the system (10).

The presence of diabetes was established based on an affirmative response to the question “Has a doctor ever told you that you have diabetes?” Health behaviors were evaluated through questions about food consumption, alcohol intake, and physical activity (Table 1). The analysis had 4 risk behaviors (regular consumption of soft drinks and sugar-sweetened beverages [≥5 days/week], consumption of meats with excess fat, consumption of milk with whole fat content, and abusive consumption of alcoholic beverages [≥4 drinks for women or ≥5 drinks for men in a single day]) and 7 protective ones (regular consumption of beans [≥5 days/week], regular consumption of fruits [≥5 days/week], regular consumption of vegetables [≥5 days/week], regular consumption of fruits and vegetables [≥5 days/week], recommended intake of fruits and vegetables [≥5 portions/day on ≥5 days/week], leisure-time physical activity [≥150 minutes/week (moderate intensity) or 75 minutes (vigorous intensity)], and transport-related physical activity [≥150 minutes/week (moderate intensity)]). Because of changes in Vigitel’s questionnaire, indicators were not necessarily available for the entire period of study (Table 1).

**Table 1:** Risk and Protective Behaviors Analyzed and Period for Which the Indicator Is Available in Vigitel,^a^ Brazil, 2006–2016

Indicator	Definition	Available for the Period
**Risk factors**		
Regular consumption of soft drinks and sugar-sweetened beverages	Consumption of beverages (soft drinks or artificial juice) with sugar on ≥5 days of the week	2007 through 2011
Consumption of meats with excess fat	Consumption of meats with apparent fat and/or chicken with skin on ≥1 days of the week	2007 through 2016
Consumption of whole fat milk	Consumption of milk with full fat content on ≥1 days of the week	2007 through 2016
Abusive consumption of alcoholic beverages	Abusive consumption in the last 30 days (≥5 drinks [men] or ≥4 drinks [women]) of alcohol in a single day	2006 through 2016
**Protective factors**		
Regular consumption of beans	Consumption of beans on ≥5 days of the week	2007 through 2016
Regular consumption of fruits	Consumption of fruits on ≥5 days of the week	2008 through 2016
Regular consumption of vegetables	Consumption of vegetables on ≥5 days of the week	2008 through 2016
Regular consumption of fruits and vegetables	Consumption of fruits and vegetables on ≥5 days of the week	2008 through 2016
Recommended intake of fruits and vegetables	Consumption of at least 5 portions of fruits and vegetables per day	2008 through 2016
Leisure-time physical activity	Leisure-time physical activity for at least 150 minutes/week (moderate intensity) or 75 minutes (vigorous intensity)	2009 through 2016
Transport-related physical activity	Transport-related physical activity for at least 150 minutes/week (moderate intensity)	2009 through 2016

a Surveillance System of Risk and Protection Factors for Chronic Diseases by Telephone Survey.

A set of sociodemographic variables complemented the analysis: sex (male or female), age group (18–24, 25–34, 35–44, 45–54, 55–64, and ≥65 years), and schooling level (0–8, 9–11, and ≥12 years of schooling).

### Statistical methods

The prevalence of diabetes (and 95% CI) was reported for each year, for the entire population by sex, age, and schooling level. The trend was evaluated by using linear regression models, with diabetes prevalence as the dependent variable and survey year as the independent variable. The regression coefficient (β) of these models indicates the average annual rate of increase or decrease of the indicator in the period, expressed in percentage points per year (pp/year). We considered the regression coefficient significant when *P* was less than .05.

The frequency of each health behavior indicator, polling the entire period for which the indicator was available, was estimated and compared between people with and without diabetes for the entire population and by sex. Poisson regression models were used to investigate the differences between people with and without diabetes, adjusting for the sociodemographic characteristics of the population (sex, age, and schooling).

We used Stata statistical software (version 13.1, StataCorp LLC) to organize the data and to perform all analyses, taking into account the Vigitel sample design.

Vigitel was approved by the National Commission for Research Ethics (protocol number 355,590) of the Ministry of Health. Consent was obtained through the verbal consent of the interviewee at the time of the interview. The study databases are freely available through the DATASUS website (Data Management Department of Brazil’s Unified Health System; www.datasus.gov.br).

## Results

The studied population consisted of 572,437 adults (≥18 years) from the capitals of Brazilian states and the Federal District, the majority being female, young adults (aged 25–44 years), and with lower schooling levels (0–11 years). However, when evaluating the sociodemographic composition trend (2006–2016) of this population, we observed that the distribution between sexes was similar, whereas the distribution according to age and schooling changed considerably. The percentage of people aged 18 to 24 years decreased from 18.9% to 14.8%, whereas the percentage of people at the opposite end of the age groups increased from 15.8% to 17.4% (45–54 years), from 10.0% to 12.3% (55–64 years), and from 9.4% to 10.9% (≥65 years). Similarly, the percentage of people with 0 to 8 years of schooling decreased from 45.5% to 32.5% and the percentage of those with 12 years of schooling or more increased from 21.2% to 31.6%.

The prevalence of diabetes increased significantly in Brazilian state capitals and the Federal District between 2006 and 2016, from 5.5% (95% CI, 5.1%–5.9%) to 8.9% (95% CI, 8.5%–9.4%) (0.28 pp/year, *P* <.001) (Table 2 and Table 3). A similar scenario was observed in both sexes, with prevalence higher in women than men. Significant increases were also identified among people aged 35 to 44 years (from 2.9% to 5.2%), 45 to 54 years (from 7.1% to 11.0%), 55 to 64 years (from 15.7% to 19.6%), and 65 years or older (from 18.9% to 27.2%). Furthermore, significant increases were seen between 2006 and 2016 at all schooling levels, highlighting the prevalence among those with lower schooling levels (0–8 years) from 8.8% to 16.5% over the study period.

**Table 2:** Distribution^a^ of Self-Reported Diabetes Mellitus in Adults (≥18 Years) From the Brazilian Capitals and Federal District by Age, Vigitel,^b^ Brazil, 2006–2016

Year (n)	Diabetes, % (95% Confidence Interval)						
	Age Group, y						Total
	18–24	25–34	35–44	45–54	55–64	≥65	
**2006 (52,796)**	0.9 (0.5–1.3)	1.1 (0.6–1.7)	2.9 (2.3–3.6)	7.1 (6.0–8.2)	15.7 (13.6–17.8)	18.9 (17.0–20.8)	5.5 (5.1–5.9)
**2007 (55,824)**	0.7 (0.4–0.9)	1.7 (1.2–2.1)	2.9 (2.3–3.4)	7.7 (6.6–8.8)	15.8 (13.8–17.8)	18.9 (17.0–20.9)	5.8 (5.4–6.2)
**2008 (54,353)**	0.6 (0.3–0.9)	0.9 (0.6–1.2)	3.4 (2.7–4.2)	9.0 (7.7–10.4)	15.7 (13.8–17.6)	21.2 (19.1–23.3)	6.2 (5.8–6.6)
**2009 (54,367)**	0.7 (0.4–0.9)	1.9 (1.0–2.7)	3.3 (2.5–4.0)	7.4 (6.3–8.5)	15.3 (13.4–17.2)	22.5 (20.2–24.7)	6.3 (5.9–6.8)
**2010 (54,339)**	1.3 (0.8–1.8)	2.2 (1.6–2.8)	3.4 (2.7–4.0)	8.1 (7.0–9.2)	16.4 (14.6–18.3)	21.9 (19.9–23.8)	6.8 (6.4–7.2)
**2011 (54,144)**	0.5 (0.2–0.7)	1.1 (0.7–1.4)	3.3 (2.6–4.0)	8.7 (7.6–9.8)	14.8 (13.2–16.5)	21.4 (19.5–23.3)	6.3 (5.9–6.7)
**2012 (45,448)**	0.9 (0.4–1.3)	1.6 (1.1–2.1)	3.9 (3.0–4.9)	9.3 (8.0–10.6)	18.5 (16.6–20.4)	22.9 (20.9–25.0)	7.4 (6.9–7.8)
**2013 (52,929)**	0.8 (0.3–1.3)	1.2 (0.8–1.6)	3.6 (2.8–4.4)	8.5 (7.3–9.7)	17.1 (15.2–18.9)	22.1 (20.4–23.8)	6.9 (6.5–7.3)
**2014 (40,853)**	1.0 (0.4–1.6)	1.6 (1.0–2.1)	3.9 (3.0–4.9)	11.5 (9.9–13.0)	18.2 (16.2–20.1)	24.4 (22.4–26.5)	8.0 (7.5–8.5)
**2015 (54,174)**	0.9 (0.5–1.2)	1.4 (0.9–1.9)	5.0 (3.9–6.1)	9.2 (7.7–10.7)	15.8 (14.2–17.5)	22.6 (20.8–24.3)	7.4 (6.9–7.9)
**2016 (53,210)**	0.9 (0.5–1.4)	2.0 (1.4–2.6)	5.2 (4.1–6.3)	11.0 (9.6–12.4)	19.6 (17.9–21.2)	27.2 (25.5–28.9)	8.9 (8.5–9.4)
**Coefficient^c^**	0.02	0.03	0.20	0.33	0.30	0.60	0.28
***P* value**	.04	.51	<.001	.004	.03	.001	<.001
**SE^d^**	0.02	0.04	0.03	0.08	0.11	0.12	0.04
***R^2^* e**	–0.02	–0.06	0.77	0.58	0.36	0.71	0.82

a Adjusted values to match the estimated total population of each city for each of the study years. For more details, see Methods section.

b Surveillance System of Risk and Protection Factors for Chronic Diseases by Telephone Survey.

c Corresponding to the linear regression coefficient value of the indicator on the year of the survey, expressed by percentage points (pp) per year. See Methods section for more details.

d Standard errors of β regression coefficient. See Methods section for more details.

e Adjusted *R^2^* corresponding to the linear regression. See Methods section for more details.

**Table 3:** Distribution^a^ of Self-Reported Diabetes Mellitus in Adults (≥18 Years) From the Brazilian Capitals and Federal District by Sex and Years of Schooling, Vigitel,^b^ Brazil, 2006–2016

Year (n)	Sex, % (95% CI)		Years of Schooling, % (95% CI)			Total
	Men	Women	0–8	9–11	≥12	
**2006 (52,796)**	4.6 (4.0–5.2)	6.3 (5.7–6.8)	8.8 (8.0–9.6)	2.8 (2.4–3.2)	2.8 (2.2–3.3)	5.5 (5.1–5.9)
**2007 (55,824)**	5.4 (4.8–5.9)	6.2 (5.7–6.7)	8.8 (8.0–9.5)	3.6 (3.1–4.1)	3.0 (2.5–3.6)	5.8 (5.4–6.2)
**2008 (54,353)**	5.7 (5.0–6.3)	6.7 (6.2–7.2)	10.3 (9.4–11.1)	3.4 (3.0–3.8)	2.6 (2.2–3.0)	6.2 (5.8–6.6)
**2009 (54,367)**	5.8 (5.1–6.5)	6.7 (6.1–7.4)	10.6 (9.6–11.6)	3.4 (3.0–3.7)	3.1 (2.7–3.5)	6.3 (5.9–6.8)
**2010 (54,339)**	6.1 (5.4–6.7)	7.4 (6.8–7.9)	10.4 (9.5–11.2)	4.6 (4.0–5.1)	4.0 (3.4–4.6)	6.8 (6.4–7.2)
**2011 (54,144)**	5.9 (5.3–6.5)	6.6 (6.1–7.1)	10.6 (9.7–11.4)	3.9 (3.4–4.3)	3.1 (2.7–3.6)	6.3 (5.9–6.7)
**2012 (45,448)**	6.5 (5.8–7.2)	8.1 (7.5–8.8)	12.1 (11.1–13.1)	5.2 (4.6–5.7)	3.8 (3.1–4.4)	7.4 (6.9–7.8)
**2013 (52,929)**	6.5 (5.8–7.2)	7.2 (6.7–7.7)	12.2 (11.3–13.2)	4.2 (3.7–4.6)	3.2 (2.8–3.7)	6.9 (6.5–7.3)
**2014 (40,853)**	7.3 (6.5–8.1)	8.7 (8.0–9.4)	14.2 (13.1–15.4)	5.1 (4.5–5.7)	3.7 (3.2–4.3)	8.0 (7.5–8.5)
**2015 (54,174)**	6.9 (6.2–7.6)	7.8 (7.2–8.4)	13.5 (12.3–14.7)	4.4 (4.0–4.9)	3.7 (3.2–4.2)	7.4 (6.9–7.9)
**2016 (53,210)**	7.8 (7.1–8.5)	9.9 (9.2–10.5)	16.5 (15.3–17.7)	5.9 (5.4–6.5)	4.6 (4.1–5.2)	8.9 (8.5–9.4)
**Coefficient^c^**	0.26	0.29	0.68	0.24	0.14	0.28
***P* value**	<.001	.001	<.001	.001	.004	<.001
**SE^d^**	0.03	0.06	0.08	0.05	0.04	0.04
***R^2^* e**	0.91	0.70	0.88	0.69	0.58	0.82

Abbreviation: CI, confidence interval.

a Adjusted values to match the estimated total population of each city for each of the study years. For more details, see Methods section.

b Surveillance System of Risk and Protection Factors for Chronic Diseases by Telephone Survey.

c Corresponding to the linear regression coefficient value of the indicator on the year of the survey, expressed by percentage points (pp) per year. See Methods section for more details.

d Standard errors of β regression coefficient. See Methods section for more details.

e Adjusted *R^2^* corresponding to the linear regression. See Methods section for more details.

In the adjusted analyses, the regular and recommended consumption of fruits and vegetables were 5% (adjusted prevalence ratio [PRa], 1.05 [*P =* .001]) and 13% (PRa, 1.13 [*P* <.001]) higher, respectively, among people with diabetes compared to those without diabetes (Table 4). Conversely, consumption of the following items was lower in people with diabetes than those without: meats with excess fat (5%) (PRa, 0.95 [*P* = .012]), whole fat milk (13%) (PRa, 0.87 [*P* <.001]), and soft drinks and sugar-sweetened beverages (43%) (PRa, 0.57 [*P* <.001]). People with diabetes also reported less abusive alcohol consumption (20%) (PRa, 0.80 [*P* <.001]), less leisure-time physical activity (8%) (PRa, 0.92 [*P* <.001]), and less transport-related physical activity (16%) (PRa, 0.84 [*P* <.001]) than those without diabetes (Table 4).

**Table 4:** Indicators of Food Consumption, Abusive Consumption of Alcoholic Beverages, and Leisure-Time Physical Activity, According to the Presence of Diabetes Mellitus, Vigitel,^a^ Brazil, 2006–2016

Indicator	Without Diabetes	With Diabetes	PRc	*P* Value	PRa^b^	*P* Value
	% (95% CI)					
**Total**						
**Food consumption**						
Fruit and vegetables ≥5 days/week	34.0 (33.8–34.3)	40.7 (39.7–41.8)	1.20	<.001	1.05	.001
Fruit ≥5 days/week	57.3 (57.0–57.6)	68.0 (67.1–69.0)	1.19	<.001	1.07	<.001
Vegetables ≥5 days/week	49.4 (49.1–49.7)	52.8 (51.8–53.8)	1.07	<.001	1.02	.12
Fruit and vegetables, >5 servings/day on ≥5 days/week	22.1 (21.9–22.4)	26.9 (25.9–27.8)	1.21	<.001	1.13	<.001
Beans ≥5 days/week	65.8 (65.5–66.0)	64.8 (63.8–65.7)	0.99	.051	0.99	.42
Meats with excess fat	32.2 (31.9–32.5)	24.3 (23.3–25.2)	0.75	<.001	0.95	.012
Whole fat milk	55.4 (55.1–55.7)	44.5 (43.5–45.5)	0.80	<.001	0.87	<.001
Soft drinks and sugar-sweetened beverages ≥5 days/week	25.0 (24.6–25.4)	9.5 (8.5–10.5)	0.38	<.001	0.57	<.001
**Abusive consumption of alcoholic beverages^c^**	17.9 (17.7–18.1)	9.0 (8.4–9.6)	0.50	<.001	0.80	<.001
**Physical activity**						
Leisure-time physical activity^d^	34.6 (34.3–34.9)	24.0 (23.1–25.0)	0.69	<.001	0.92	<.001
Transport-related physical activity^e^	13.8 (13.6–14.0)	8.8 (8.1–9.4)	0.64	<.001	0.84	<.001
**Men**						
**Food consumption**						
Fruit and vegetables ≥5 days/week	27.5 (27.1–27.9)	35.0 (33.3–36.7)	1.27	<.001	1.12	<.001
Fruit ≥5 days/week	50.9 (50.4–51.3)	62.0 (60.3–63.6)	1.22	<.001	1.11	<.001
Vegetables ≥5 days/week	43.6 (43.1–44.0)	48.1 (46.4–49.7)	1.10	<.001	1.05	.007
Fruit and vegetables, >5 servings/day on ≥5 days/week	17.7 (17.3–18.1)	22.2 (20.7–23.7)	1.25	<.001	1.17	<.001
Beans ≥5 days/week	72.3 (71.9–72.7)	71.4 (70.0–72.9)	0.99	.247	1.00	.94
Meats with excess fat	42.9 (42.5–43.4)	34.2 (32.5–36.0)	0.80	<.001	0.94	.03
Whole fat milk	58.0 (57.5–58.5)	47.3 (45.6–49.1)	0.82	<.001	0.89	<.001
Soft drinks and sugar-sweetened beverages ≥5 days/week	29.3 (28.7–29.9)	9.8 (8.3–11.4)	0.34	<.001	0.49	<.001
**Abusive consumption of alcoholic beverages^c^**	26.8 (26.4–27.2)	15.9 (14.7–17.1)	0.59	<.001	0.86	<.001
**Physical activity**						
Leisure-time physical activity^d^	43.2 (42.6–43.7)	27.5 (25.9–29.1)	0.64	<.001	0.90	.001
Transport-related physical activity^e^	14.6 (14.2–14.9)	9.7 (8.6–10.7)	0.66	<.001	0.82	.001
**Women**						
**Food consumption**						
Fruit and vegetables ≥5 days/week	39.7 (39.3–40.1)	44.9 (43.5–46.2)	1.13	<.001	1.01	.61
Fruit ≥5 days/week	62.9 (62.5–63.2)	72.4 (71.2–73.5)	1.15	<.001	1.04	<.001
Vegetables ≥5 days/week	54.4 (54.1–54.8)	56.2 (55.0–57.5)	1.03	.004	0.99	.49
Fruit and vegetables, >5 servings/day on ≥5 days/week	26.0 (25.6–26.3)	30.2 (29.0–31.4)	1.16	<.001	1.11	<.001
Beans ≥5 days/week	60.1 (59.7–60.4)	60.0 (58.7–61.2)	1.00	.847	0.99	.27
Meats with excess fat	22.9 (22.6–23.3)	17.1 (16.1–18.1)	0.74	<.001	0.95	.14
Whole fat milk	53.1 (52.7–53.5)	42.5 (41.2–43.7)	0.80	<.001	0.85	<.001
Soft drinks and sugar-sweetened beverages ≥5 days/week	21.3 (20.9–21.8)	9.2 (7.9–10.5)	0.43	<.001	0.64	<.001
**Abusive consumption of alcoholic beverages^c^**	10.3 (10.0–10.5)	4.0 (3.5–4.5)	0.39	<.001	0.68	<.001
**Physical activity**						
Leisure-time physical activity^d^	27.2 (26.8–27.5)	21.5 (20.4–22.7)	0.79	<.001	0.96	.12
Transport-related physical activity^e^	13.1 (12.8–13.4)	8.1 (7.3–8.8)	0.62	<.001	0.86	.003

Abbreviations: CI, confidence interval; PRa, adjusted prevalence ratio; PRc, crude prevalence ratio.

a Surveillance System for Risk and Protection Factors for Chronic Diseases by Telephone Survey.

b Adjustment variables: age and schooling.

c ≥5 drinks for men and ≥4 for women in a day.

d ≥150 minutes/week of moderate-intensity activity or ≥75 minutes of vigorous activity.

e ≥150 minutes/week of moderate-intensity activity.

More protective behaviors and fewer risk behaviors were observed in men and women with diabetes than those without (Table 4). Men with diabetes had a higher regular (12%) (PRa, 1.12 [*P* <.001]) and recommended (17%) (PRa, 1.17 [*P* <.001]) consumption of fruits and vegetables and lower consumption of meats with excess fat (6%) (PRa, 0.94 [*P =* .03]), whole fat milk (11%) (PRa, 0.89 [*P* <.001]), soft drinks and sugar-sweetened beverages (51%) (PRa, 0.49 [*P* <.001]), and abusive consumption of alcoholic beverages (14%) (PRa, 0.86 [*P* <.001]) than those without diabetes. Women with diabetes also presented more favorable health behaviors when compared to women without diabetes (Table 4).

However, women with diabetes tended to have higher frequencies of protective factors and lower frequencies of risk factors compared to men with diabetes, such as regular consumption of fruits and vegetables (44.9% vs 35.0%), recommended consumption of fruits and vegetables (30.2% vs 22.2%), consumption of meats with excess fat (17.1% vs 34.2%), consumption of whole fat milk (42.5% vs 47.3%), and the abusive consumption of alcoholic beverages (4.0% vs 15.9%) (Table 4). Regarding leisure-time physical activity, a significant difference was identified only among men (10% lower in men with diabetes compared to those without diabetes; PRa, 0.90 [*P* = .001]). The prevalence of transport-related physical activity was lower in both men and women with diabetes (PRa, 0.82 [*P* = .001] and PRa, 0.86 [*P* = .003], respectively) in comparison to those without diabetes (Table 4).

## Discussion

Information acquired in 572,437 interviews conducted by Vigitel in 2006 through 2016 allowed us to investigate the evolution of diabetes prevalence and health behaviors in the adult Brazilian population. The results show an increase in diabetes prevalence between 2006 and 2016, mainly among women, older adults, and those with less than a high school education. These groups also had the highest diabetes prevalence at the beginning of the study period. In general, people with diabetes had a lower frequency of health risk behaviors and a higher frequency of health protective behaviors when compared to people without diabetes. A significant increase in diabetes prevalence was observed even for the population educated beyond high school, who are generally recognized as having a lower risk of adopting risky behaviors. The increase in diabetes among a more highly educated population indicates that the economic development and urbanization experienced in the last decades affected lifestyle in terms of risk factors of diabetes (2).

Behavioral health factors for people with diabetes have already been investigated in population surveys from different developed nations such as Australia (12) and France (13). However, until this study, no similar investigation was found for large samples of people with diabetes in developing countries. The findings of this study are aligned with those from populations in developed countries, indicating better food consumption among people with diabetes when compared to those without diabetes (13,14), especially regarding higher consumption of fruits and vegetables (13,14), lower consumption of soft drinks and sweets (14), and lower abusive consumption of alcoholic beverages (13,14).

Our results show healthier lifestyles among women, both with and without diabetes, expanding the current evidence for women’s greater adherence to health-protective behaviors (15). In Brazil, the incidence and risk factors for noncommunicable diseases by sex were investigated by the cohort study, Brazilian Longitudinal Study of Adult Health. A greater proportion of women were classified as having a healthier lifestyle (no current tobacco use, no moderate or excessive alcohol consumption, frequent physical activity, and healthy eating) when compared with men, which reinforces the findings in our study (15). This finding can be explained by greater access of Brazilian women (with or without noncommunicable diseases) to health services and medical consultations than men (16).

Despite the healthier food consumption of people with diabetes when compared to those without diabetes, both groups still showed a high frequency of negative behaviors. Almost half of the people with diabetes reported consuming whole fat milk and 1 in 10 reported regularly consuming sugar-sweetened beverages. In addition, most people with diabetes did not achieve the regular and recommended consumption of fruits and vegetables (59.3% and 73.1%, respectively). Longitudinal studies have already demonstrated the importance of adequate health-related behaviors to reduce mortality and complications from noncommunicable diseases (17). The low prevalence of physical activity among people with diabetes identified in our study aligns people with diabetes in Brazil to those living in countries such as the United States (18) and Portugal (19). The benefits of physical activity in the prevention or management of diabetes are already well established (20), reducing mortality and complications through better glycemic control and improved insulin sensitivity.

In Brazil, much of the advice promoting healthy eating and physical activity for people with diabetes is carried out in primary health care services (4,21), in a strategy similar to that adopted in countries with comprehensive health systems such as Canada (22) and Australia (23). Specific protocols for the management of diabetes, such as *Estratégias para o cuidado da pessoas com doença crônica: Diabetes Mellitus* (*Strategies for Care of People With Chronic Disease: Diabetes Mellitus*) (4) and the strategic plan to tackle noncommunicable diseases, *Plano de Ações Estratégicas para o Enfrentamento das Doenças Crônicas Não Transmissíveis (DCNT) no Brasil, 2011–2022* (21), reinforce the importance of our findings by proposing actions and targets to control noncommunicable diseases. Our results highlight the need for greater planning and promotion of healthy eating among people with diabetes in Brazil, with special attention to reducing consumption of sugar-sweetened beverages and increasing consumption of fruits and vegetables.

Regarding physical activity promotion, the Ministry of Health launched the Health Academy Program (*Academia da Saúde*) in 2011. This program provides free public gyms for the population in the major cities of the country; these gyms are the main action used to promote physical activity in the context of primary health care (24). Insufficient levels of physical activity among people with diabetes demonstrated in our study reinforce the importance of expanding these actions.

This is the first population-based study investigating health behaviors among people with and without diabetes in Brazil. Our evidence strengthens the hypothesis that people with diabetes are more likely to adhere to healthy lifestyles than people without diabetes, an important issue in improving quality of life and disease control among people with diabetes (25).

However, this does not mean that people with diabetes correctly follow all guidelines for better management of the disease. Our hypothesis is that after receiving a diagnosis of diabetes, counseling for lifestyle changes in primary care has led these people to healthier habits. However, these actions are still insufficient because most people with diabetes did not attain the regular and recommended consumption of fruits and vegetables or sufficient physical activity.

The same scenario can occur in developing countries with similar sociodemographic characteristics, because these factors influence the magnitude of disease in the population. Aspects such as urbanization, modernization, and more women having jobs have modified Latinos’ eating habits, with access to a wide variety of ultraprocessed products (26). In Colombia, for example, the death rate attributable to diabetes was higher among women than men (26), and in Mexico diabetes prevalence will continue to increase even if current incidence rates remain unchanged (27). Thus, it is expected that diabetes will continue to have a prominent place in a population’s epidemiologic profile (3).

Our study has some limitations. The data presented on diabetes frequency involve only people who reported a medical diagnosis of diabetes and do not include people with as yet undiagnosed diabetes. A substantial proportion of diabetes cases remain undiagnosed because of a lack of clinical manifestations of the disease combined with poor access to health services (28). Thus, the values shown here cannot be seen directly as the diabetes prevalence in the country but only an approximation of it. However, this issue does not play a major role in comparisons of health behaviors between groups with and without diabetes. Other limitations of this study include restricting the sample to people who had a landline telephone and using self-reported information to estimate diabetes diagnosis, food consumption, abusive consumption of alcoholic beverages, and physical activity. To account for differences in the composition of the population with and without landline telephones, Vigitel uses weighting factors that allow adjustment of the estimates and extrapolation of the results to the total population of the locations investigated (10). Additionally, the self-reported diagnosis of diabetes and different health behaviors has been widely used in large surveys (29) and found to be valid and reliable in studies conducted with the Brazilian population (30,31).

The increase in diabetes prevalence in recent years raises concerns related to the status of this disease as a public health problem. Although individuals with diabetes have, in general, better indicators of eating behavior than the rest of the population, they still have a frequency of bad eating habits beyond what is desirable as well as insufficient physical activity. Because most behavioral risk factors of diabetes are also related to complex disease management and adverse prognoses, the ideal public health scenario would involve a low frequency of risk factors both in the population with diabetes (for adequate management of the disease) and in those without it (aimed at primary prevention).
